# Early prediction of treatment response to high-dose salvage chemotherapy in patients with relapsed germ cell cancer using [^18^F]FDG PET

**DOI:** 10.1038/sj.bjc.6600122

**Published:** 2002-02-12

**Authors:** C Bokemeyer, C Kollmannsberger, K Oechsle, B M Dohmen, A Pfannenberg, C D Claussen, R Bares, L Kanz

**Affiliations:** Department of Hematology/Oncology, University of Tuebingen Medical Center, Otfried-Mueller-Str 10, 72076 Tuebingen, Germany; Department of Nuclear Medicine, University of Tuebingen Medical Center, Otfried-Mueller-Str 10, 72076 Tuebingen, Germany; Department of Radiology, University of Tuebingen Medical Center, Otfried-Mueller-Str 10, 72076 Tuebingen, Germany

**Keywords:** germ cell cancer, response monitoring, PET, tumour markers, high-dose chemotherapy

## Abstract

To assess the ability of [^18^F]fluorodeoxyglucose positron emission tomography for the early prediction of response in patients with relapsed metastatic germ cell tumours undergoing salvage high-dose chemotherapy. The role of positron emission tomography was compared with established means of tumour response assessment such as CT scans/MRI and serum tumour marker changes. In addition, positron emission tomography was compared with a current prognostic score which differentiates three prognostic groups with failure-free survival rates ranging from 5–50%. [^18^F]fluorodeoxyglucose uptake of metastases from germ cell tumours as well as CT scans and serum tumour marker were acquired after 2–3 cycles of induction chemotherapy but before the start of high-dose chemotherapy and CT scans/serum tumour marker were compared with the baseline examinations in 23 patients with relapsed germ cell tumours. To evaluate the validity of early response prediction by positron emission tomography, radiological monitoring and serum tumour marker decline, histopathologic response after resection of residual masses and/or the clinical course over 6 months after the end of treatment (relapse *vs* freedom of progression) were used. Overall, 10 patients (43%) achieved a marker-negative partial remission, three (13%) a marker-positive partial remission, five (22%) a disease stabilization and five (22%) progressed during treatment. Nine patients (39%) remained progression-free over 6 months following treatment, whereas 14 (61%) progressed. The outcome of high-dose chemotherapy was correctly predicted by positron emission tomography/CT scan/serum tumour marker in 91/59/48%. Eight patients with a favourably predicted outcome by CT scans plus serum tumour marker but a positive positron emission tomography prior to high-dose chemotherapy, failed treatment. This results in the following sensitivities/specificities for the prediction of failure of high-dose chemotherapy: positron emission tomography 100/78%; radiological monitoring 43/78%; serum tumour marker 15/100%. The positive and negative predictive values of positron emission tomography were 88 and 100%, respectively. As compared with the prognostic score, positron emission tomography was correctly positive in all patients of the three risk groups who failed treatment. In addition, a negative positron emission tomography correctly predicted a favourable outcome in the good and intermediate group. [^18^F]fluorodeoxyglucose positron emission tomography imaging can be used to assess response to chemotherapy in patients with relapsed germ cell tumours early in the course of treatment and may help to identify patients most likely to achieve a favourable response to subsequent high-dose chemotherapy. In patients with response to induction chemotherapy according to CT scans or serum tumour marker evaluation, positron emission tomography seems to add information to detect patients with an overall unfavourable outcome. It may also be a valuable addition to the prognostic model particularly in the good and intermediate group for further selection of patients who will profit from high-dose chemotherapy.

*British Journal of Cancer* (2002) **86**, 506–511. DOI: 10.1038/sj/bjc/6600122 www.bjcancer.com

© 2002 Cancer Research UK

## 

The development of cisplatin-based combination chemotherapy has dramatically improved the prognosis of patients with metastatic germ cell cancer, resulting in a long-term cure rate of 70–80% (Einhorn, 1990; Hartmann *et al*, 1999). However, patients relapsing after cisplatin-based first-line chemotherapy exhibit unsatisfactory survival rates of only 20–30% following standard-dose cisplatin-based salvage chemotherapy (Rick *et al*, 1999). In these patients, high-dose chemotherapy (HD-CTX) with autologous hematopoietic stem cell support has been widely investigated in order to improve their outcome. Studies have suggested a potential survival improvement of 10–20% using HD-CTX, but this survival increase has not yet been confirmed by a randomized trial (Broun *et al*, 1995; Motzer *et al*, 1996; Beyer *et al*, 1999; Rick *et al*, 2001). However, HD-CTX is associated with more frequent side effects as well as higher financial costs compared with standard-dose chemotherapy. Thus, it is of great interest, whether one can identify early those patients who will benefit from HD-CTX and those who will not (Anthoney and Kaye, 1999; Decartis *et al*, 2000).

Clinical prognostic factors for salvage chemotherapy have been proposed in order to identify those patients with a very poor prognosis despite the use of salvage HD-CTX and distinguish them from patients with an intermediate or good chance for success of HD-CTX. These unfavourable prognostic factors include high ß-HCG levels, disease refractory to platin-based chemotherapy, primary mediastinal tumour and disease progression prior to HD-CTX (Beyer *et al*, 1996). Based on these factors, patients can be classified prior to the start of salvage therapy into three risk groups, good, intermediate and poor, with a likelihood of 2 year failure-free survival of 51, 27 and 5%, respectively.

For assessing the tumour response during treatment, both the timely decline of tumour markers and the documentation of morphologic changes in tumour size by radiological methods such as CT scan or MRI have been used up to date. However, the decline of serum tumour markers depends on certain metabolic pathways, which may be of different capacity in individual patients (deWit *et al*, 1997; Motzer *et al*, 1997; Zon *et al*, 1998; Christensen *et al*, 1999). Prediction of response during chemotherapy by tumour marker decline alone may be misleading. Radiological imaging for the assessment of tumour response may also be of limited value due to the delay between the start of treatment and tumour shrinkage and due to the inability of radiological imaging techniques to differentiate vital carcinoma from necrosis or scar tissue in case of residual lesions. Thus, additional means for the monitoring of treatment response may be helpful to identify non-responding patients and to avoid ineffective and toxic treatment.

Positron emission tomography (PET) imaging using 2-[^18^F]fluoro-2-deoxy-D-glucose (F-18 FDG) is a new diagnostic technique which allows visualization and quantification of regional glucose metabolism within the body (Strauss and Conti, 1991; Hawkins *et al*, 1992). Since cancer cells are characterized by a higher glucolytic rate than normal tissue cells, PET exploits this difference by assessing the rate and quantity of F-18 FDG uptake by the tumour. Studies have investigated the role of PET for the evaluation of residual masses in patients with germ cell tumours (GCT) after chemotherapy demonstrating the additional value of PET imaging for the detection of viable carcinoma in residual masses of both patients with seminomatous and non-seminomatous germ cell cancer (Stephens *et al*, 1996; Cremerius *et al*, 1998; Ganjoo *et al*, 1999; De Santis *et al*, 2001; Kollmannsberger *et al*, 2002). However, no study has so far reported the ability of PET to predict response during standard-dose or HD-CTX in patients with germ cell cancer.

Thus, the primary aim of the present prospective study was to evaluate the general ability of PET to predict response to salvage chemotherapy in patients with relapsed metastatic germ cell cancer. In-vivo chemotherapy sensitivity testing by PET was correlated with the overall outcome of the patient following subsequent HD-CTX. In addition, PET was compared with the above described prognostic score for high-dose salvage chemotherapy (Beyer *et al*, 1996) as well as to the tumour marker decline and assessment radiological imaging, which are the currently established methods for assessment of response to chemotherapy

## MATERIALS AND METHODS

### Patients and treatment

Patients with relapsed disease after cisplatin-based first-line chemotherapy participating in the prospective German multicenter HD-CTX trial between September 1995 and October 1999 were eligible for inclusion into the PET protocol (Mead for the International Germ Cell Cancer Collaborative Group, 1997; Rick *et al*, 2001). All patients were treated at Tuebingen University Medical Center. Eligibility criteria for high-dose salvage chemotherapy consisted of the following: relapsed GCT of any primary tumour site after first-line chemotherapy treatment, Karnofsky performance status >50%, normal kidney function, absence of severe heart or liver disease and written informed consent. Patients were treated with three cycles of standard-dose TIP-chemotherapy followed by one cycle of TEC-HD-CTX. Standard-dose TIP-chemotherapy consisted of paclitaxel 175 mg m^−2^ given on day 1 and ifosfamide 1200 mg m^−2^ and cisplatin 20 mg m^−2^ both administered daily on days 2 through 6 of a 22-days cycle. The TEC-high-dose regimen contained thiotepa 150 mg m^−2^, etoposide 600 mg m^−2^ and carboplatin 500 mg m^−2^, all drugs given daily over 3 days. All patients received autologous peripheral blood stem cell support and granulocyte-colony stimulating factor after HD-CTX according to treatment protocol and institutional practice. The results of this trial have been previously published (Rick *et al*, 2001).

All patients were treated at Tuebingen University Medical Center. Both studies, the HD-CTX trial as well as the present PET study were approved by the Ethical Committee of the University of Tuebingen. All patients were required to provide informed consent before study entry.

### Tumour response evaluation

**Figure 1:**
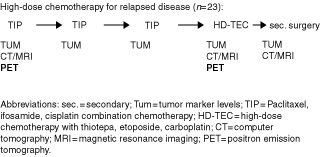
Schedule of CT/MRI and PET examinations for response assessment during chemotherapy according to the protocol.

All patients underwent extensive staging procedures including CT scan or MRI of the chest, abdomen, and brain, determination of serum tumour marker (TUM) levels (ß-HCG, AFP, LDH) as well as a baseline PET imaging prior to the start of chemotherapy. All staging procedures were performed within 5 days in order to allow comparisons. Determination of TUM levels were repeated prior to each of the subsequent chemotherapy cycles and CT scans and/or MRI of the tumour lesions after every second cycle. Induction chemotherapy included the three standard-dose chemotherapy cycles. Within 8 days prior to the planned high-dose TEC therapy, PET imaging, CT scan and/or MRI of the tumour lesions as well as a determination of TUM levels were repeated ( Figure 1

). After completion of salvage chemotherapy, all residual masses in patients with non-seminomatous germ cell cancer were resected, if technically possible. All patients underwent follow-up examinations including evaluation of tumour marker levels and CT scans in 3-monthly intervals.

All CT/MRI scans were reviewed by an independent, board-certified radiologist who was not aware of the PET findings. Response was classified according to modified WHO criteria (deWit *et al*, 1998).

A partial or complete remission on CT scan as well as normalized or declining TUM (>90% decline compared with baseline value) during chemotherapy were classified as a favourable response predicting a successful outcome. Stable disease or disease progression on CT scan or a tumour marker decrease <90% or increasing markers prior to HD-CTX were considered as an unfavourable response and thus predictive for treatment failure.

### PET imaging

A dedicated PET scanner (ADVANCE, General Electrics Medical Systems, Milwaukee, WI, USA) was used, providing an axial field of view (FOV) of 15.3 cm. Emission data were corrected for random events, attenuation and scattering. Per FOV 35 slices (4.25 mm) were reconstructed iteratively or by filtered back projection with a 128×128 pixel matrix (4.3×4.3 mm pixel size) resulting in a final resolution of about 8 mm (full width at half maximum).

All patients fasted for at least 12 h before PET imaging. Blood glucose levels were checked for each patient before the intravenous administration of 250–500 MBq of F-18 FDG. In all patients, 1–3 FOVs were imaged depending on the lesions found on CT scan. Forty-five to 60 min after the F-18 FDG injection, static PET-scans were recorded for 5–15 min per FOV (depending on reconstruction algorithm, injected radioactivity and patient weight). Transmission scanning (10–20 min resp. 500 000 kcts per FOV; sinogram windowing) was performed before F-18 FDG-injection or after emission scanning. After tracer injection, patients received at least 1 l of normal saline and 20 mg furosemide in order to minimize image artefacts from residual radioactivity in the urinary tract.

### PET image analysis

Each PET was reviewed by an experienced nuclear medicine physician, who was blinded to the TUM course as well as to the interpretation of the results of CT scan and/or MRI. Standardized uptake values (SUV), a quantitative measure of tumour uptake of the tracer, adjusted for injected dose and body weight, were calculated, if an increased uptake was observed in the tumour area (Leskinen *et al*, 1991; Strauss and Conti, 1991). Patients with a SUV of ⩾2 were considered positive (Kollmannsberger *et al*, 2002).

PET findings after induction chemotherapy were also correlated to the histological findings of the residual mass in those patients who received a secondary resection after completion of chemotherapy treatment. If no resection of residual tumour masses was performed, the clinical course of the patient was used. If no radiological tumour progression or increase of tumour markers were observed within 6 months after the end of therapy, the residual tumour was considered avital. The 6 month interval was chosen since clinically a disease-free interval of at least 6 months appears to justify the physical and financial expenses of HD-CTX with stem cell support.

## RESULTS

Twenty-three patients receiving HD-CTX for relapsed germ cell cancer were evaluated with F-18 FDG-PET imaging before the start of treatment as well as during the course of treatment. Characteristics of the patients are listed in Table 1

**Table 1:**
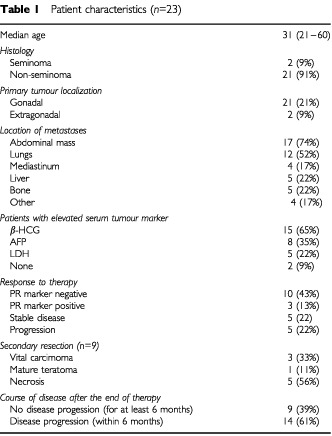
Patient characteristics (*n*=23)

. Baseline PET prior to the start of chemotherapy was positive in all lesions evaluated in this study.

In 14 patients the clinical course over 6 months was monitored while in the other nine patients the histology following secondary resection of residual masses was available. Overall, nine patients (39%) remained progression-free for at least 6 months following HD-CTX, whereas 14 (61%) patients relapsed within this time period. Median follow-up was 27 months (10–55 months) for all patients and only three patients relapsed beyond the follow-up period of 6 months after the PET examination (8, 16 and 18 months after the PET examination).

### Outcome prediction by PET

Overall, the clinical course of disease after HD-CTX was correctly predicted by PET imaging during chemotherapy in 21 of 23 patients (91%). A negative PET (SUV <2) after the initial part of treatment was found in seven patients (30%) and none of these patients failed after the completion of the full treatment regimen. In 16 patients (70%) PET was still positive prior to the actual HD-CTX cycles. Fourteen of these patients either relapsed within 6 months following HD-CTX or the histology of the resected residual tumour mass after HD-CTX still revealed the presence of vital carcinoma. Two patients with a positive PET (SUV ⩾2) prior to the HD-CTX still had a favourable outcome. In the first patient a SUV of 5.4 (pulmonary metastasis) and in the second patient a SUV of 7.5 (mediastinal mass) suggested the presence of gross vital carcinoma and thus poor response to induction chemotherapy. However, histology of the resected masses after the completion of therapy only showed necrosis in both patients. Thus, sensitivity and specificity of PET for the prediction of the overall failure of salvage chemotherapy are 100 and 78%, respectively. The positive predictive value of PET for treatment failure was 88%, whereas the negative predictive value of PET was 100% ( Table 2

**Table 2:**
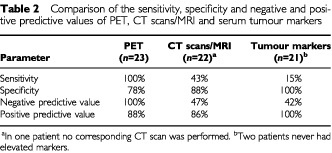
Comparison of the sensitivity, specificity and negative and positive predictive values of PET, CT scans/MRI and serum tumour markers

).

### Comparison of PET to CT scan/MRI for the prediction of therapy response

Comparisons of PET to CT scan/MRI performed at the same time point during chemotherapy were available for 22 patients. In one patient no corresponding CT scan was performed. Overall, CT scans/MRI correctly predicted the outcome after therapy in 13 (59%) patients. CT scans/MRI correctly indicated a non-responding tumour in six patients, four of whom showed stable disease and two progressive tumour lesions during chemotherapy. All of these six patients also had a positive PET during chemotherapy. Seven patients with a favourable response to HD-CTX showed regression of their metastases on CT scans/MRI after induction therapy. Only five of these patients were PET negative, whereas in two patients the PET still showed increased SUV-values prior to high-dose chemotherapy. CT scans/MRI during therapy were not able to correctly predict the outcome in nine patients. Eight of these patients showed a remission of metastases on CT scan/MRI but still had an unfavourable response. One patient remained disease-free after treatment despite the CT scans showing a stable disease prior to the HD-CTX. All of these nine patients had PET results correctly predicting the overall response, eight still being positive and one already being negative. Thus, the sensitivity and specificity of CT scans/MRI (SD or PD predicting an unfavourable outcome) were 43 and 88% respectively. The positive and negative predictive values of CT scans/MRI were 86 and 47%, respectively (Table 2).

### Comparison of PET to TUM decline for the prediction of therapy outcome

The comparison of PET to TUM decline during therapy included 21 patients since two patients never had had elevated markers. The response to HD-CTX by tumour marker decline was correctly predicted in 10 patients (48%). In two patients tumour markers remained elevated or even increased during therapy and both patients failed treatment. These two patients also showed a positive PET scan prior to HD-CTX. Tumour marker decline correctly predicted a favourable outcome in eight patients, five of whom had already normalized and three declining markers during chemotherapy. PET was also already normalized in seven of these eight patients, but in one patient PET was still positive prior to HD-CTX. The decline (nine patients) or normalization (two patients) of tumour markers indicated a favourable outcome in 11 further patients, but all of these failed treatment. In contrast, PET correctly predicted the course of disease in these 11 patients. There were no patients with a false positive elevation of tumour markers. The sensitivity and specificity of tumour marker elevation indicating treatment failure were 15 and 100%, respectively. The positive predictive value was 100% and the negative predictive value 42% (Table 2).

### Comparison of PET to the combined assessment by CT scans/MRI and serum marker decline

For the comparison of PET to the combination of CT scan/MRI plus TUM decline data from 20 patients were available. Two patients never had elevated tumour markers and in one patient no corresponding CT scan was performed at the time of PET imaging. Of 20 patients, response to treatment was correctly predicted by the combination of CT scan/MRI plus tumour marker decline in 12 patients (60%). Five patients showed a stable disease/tumour progression on CT scans/MRI as well as a persisting elevation/increase of tumour markers. All of these five patients relapsed after HD-CTX. PET imaging performed during chemotherapy was positive in these five patients. CT scans/MRI findings as well as tumour markers were improved following induction chemotherapy in seven patients correctly indicating a favourable outcome. PET was also predictive in six of these seven patients whereas in one patient the PET prior to HD-CTX was still markedly positive. However, in eight patients the combination of CT scan/MRI plus tumour marker decline suggested a favourable response to chemotherapy, while the positive PET scan during chemotherapy in all of these eight patients correctly predicted their unfavourable outcome ( Table 3

**Table 3:**
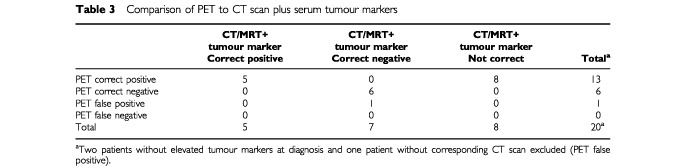
Comparison of PET to CT scan plus serum tumour markers

).

### Comparison of F-18 FDG PET with the prognostic score by Beyer *et al* (1996)

Based on the prognostic factors described by Beyer *et al* (1996) nine patients (39%) belonged to the good risk group, another nine patients (39%) to the intermediate group and the remaining five patients (22%) to the poor risk group. Three patients in the good risk group, six patients in the intermediate group and all patients in the poor risk group relapsed after salvage HD-CTX. PET performed during salvage chemotherapy was positive in all of these patients correctly predicting their unfavourable outcome. PET was correctly negative in five of six patients and in two of three patients in the good and intermediate group, respectively, who remained relapse-free after treatment. In two patients, one in the good and one in intermediate risk group, PET showed an increased F-18 FDG uptake prior to HD-CTX, suggesting an unfavourable outcome, but both patients remained relapse-free following treatment. There were no false negative PET results in any of the three groups ( Table 4

**Table 4:**
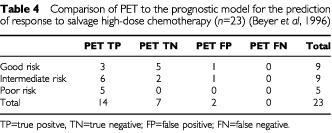
Comparison of PET to the prognostic model for the prediction of response to salvage high-dose chemotherapy (*n*=23) (Beyer *et al*, 1996)

).

## DISCUSSION

Over the last decade, HD-CTX with autologous stem cell support has been widely investigated as salvage therapy for patients with refractory or relapsed germ cell cancer (Motzer *et al*, 1996; Bhatia *et al*, 1998; Rick *et al*, 2001). Since not all patients will benefit to the same amount from this treatment, prognostic scores have been developed in order to identify those patients who are most likely to respond (Beyer *et al*, 1996) and thus, in whom the physical and financial costs of HD-CTX can be justified (Anthoney and Kaye, 1999; Decartis *et al*, 2000). It was the aim of the present study to determine whether metabolic monitoring using PET early in the course of salvage chemotherapy can be used to predict the overall response to treatment and indicate the outcome in patients with relapsed germ cell cancer. Moreover, the value of PET imaging was investigated comparing it to an established prognostic model as well as to conventional means of response assessment such as CT scans/MRI and the changes of TUM levels.

Studies in different tumour types have demonstrated the ability of PET to clinically document tumour response to chemotherapy as well as its diagnostic value in the staging of cancer (Huovinen *et al*, 1993; Findlay *et al*, 1996; Flamen *et al*, 1999; Kollmannsberger *et al*, 2002). Wahl *et al* (1993) were among the first who reported that PET may have a substantial role as an early non-invasive method for the assessment of the efficacy of treatment. Two recently published reports suggest that F-18 FDG-PET may be valuable for the prediction of response to neoadjuvant chemotherapy in patients with advanced breast cancer. Schelling *et al* (2000) demonstrated that in breast cancer patients F-18 FDG-PET can differentiate responders from non-responders already after the first cycle of neoadjuvant chemotherapy with a high rate of accuracy. Similar results were reported by Smith *et al* (2000). In patients with pancreatic cancer, PET has been reported to be superior to response assessment by conventional radiographical methods and may subsequently predict survival (Maisey *et al*, 2000).

In the current study in patients with relapsed germ cell cancer, PET performed after the initial cycles of salvage induction chemotherapy was able to correctly predict the outcome of HD-CTX in 91% of patients. A negative PET prior to HD-CTX appears to be a strong predictor for a favourable outcome, since none of these patients failed treatment. In contrast, a PET still remaining positive after the first chemotherapy cycles was highly predictive for an overall unfavourable outcome despite the use of subsequent HD-CTX. Only in two patients with an overall favourable response, PET results were still positive prior to HD-CTX. Histology revealed necrosis combined with inflammation in both cases. In these two cases, either PET may have been correctly positive and HD-CTX may have eradicated the vital tumour or PET may have been false positive due to an inflammatory process. F-18 FDG is not a tumour-specific agent and it may also accumulate in tissue macrophages. This phenomenon is a major source of false-positive PET examinations in cancer patients (Strauss, 1996; Flamen *et al*, 1999). In order to reduce the rate of false positive findings it is necessary to correlate the PET results with data from other methods for response assessment such as CT scans and the decline of previously elevated tumour markers. Considering separately the sensitivities and specificities of F-18 FDG-PET, and radiological response, our results indicate that no method by itself is sufficiently accurate to predict the overall treatment result.

To our knowledge, this is the first study which compares F-18 FDG-PET to the prognostic model based on clinical factors. This study also compares 18F-FDG PET results with established criteria for response assessment in patients with metastatic germ cell cancer. PET may be a valuable addition to the established prognostic model developed by Beyer *et al* (1996) and may be able to further select patients particularly in the good and intermediate risk group who will or will not profit from salvage HD-CTX. A positive PET prior to HD-CTX appears to be highly predictive for an unfavourable outcome despite ‘good risk’ prognostic features. In contrast, a negative PET prior to HD-CTX seems to reliably predict an favourable outcome even if the patient exhibits unfavourable prognostic characteristics.

Since this study was performed prospectively with blinded reading of CT scans/MRI, and PET, none of the diagnostic physicians was aware of the results of the corresponding tests. Thus, differences in sensitivity and specificity can be directly compared based on the results obtained in this study. As compared with CT scans/MRI and tumour markers, PET seems to offer additional information regarding treatment outcome in a large number of patients. Persisting or even increasing tumour markers as well as progressive disease on CT scan/MRI during chemotherapy are strong predictors for an unfavourable outcome of treatment. In these patients PET was not able to add additional information regarding response. However, the potential benefit of PET was observed in patients with stable disease or remissions on CT scan/MRI and/or declining or normalized tumour markers. These patients form the largest subgroup. In these patients an elevated F-18 FDG uptake correctly predicted the unfavourable outcome of eight patients in whom CT scans/MRI and the tumour marker course suggested a favourable response to therapy. One of the most important factors for the success of HD-CTX in relapsed patients is responsiveness of disease to previous platin-based therapy. Using PET as an early method of response assessment, this non-invasive technique seems to offer the chance for in-vivo chemosensitivity testing during treatment.

However, the limitations of the present study have to be considered. The number of patients included was small which might cause misleading results. Larger studies are necessary to confirm these results. A second limitation of the present study could be the methodology used to confirm the prediction of PET. Whereas it would have been ideal to have a histological diagnosis of all tumours at the time of response prediction, this was certainly not feasible during the course of chemotherapy. The prediction of response is largely based on the follow-up of the patients over 6 months after HD-CTX. Since relapse or progression of germ cell cancer, once present, usually occurs rapidly after the end of treatment, the data obtained in this study may still be valid. Most of the relapses in our study occurred within 6 months after treatment. However, three patients relapsed beyond the 6 months follow-up period indicating that PET similar to other methods of response assessment may be unable to predict for late relapses.

Our study has intentionally focused on a specific population of germ cell cancer patients who are at a high risk for incomplete response and relapse. This study population served as a model for the evaluation of the ability of PET to predict response to treatment already during the course of therapy. The high rate of non-responding/relapsing patients allows the evaluation of PET's predictive value as a ‘proof of principle’. PET performed in conjunction with clinical prognostic factors as well as conventional staging methods appears to offer additional information for response prediction during chemotherapy. Whether patients with a negative PET after three cycles of conventional-dose salvage chemotherapy benefit from subsequent HD-CTX or may have a similar favourable outcome with one additional conventional-dose chemotherapy cycle is presently unclear and remains the objective of future studies. Nevertheless, at this point in time it is not justified to derive treatment decisions from PET results alone. The predictive value of PET should now also be examined in patients with earlier stages of metastatic disease, where treatment intensification may still be a therapeutic option.

Since PET imaging performed early in the course of treatment may provide independent prognostic information, a multivariate analysis needs to investigate its impact in relation to other clinical and biological prognostic factors. Moreover, the early identification of non-responding patients by PET may help to avoid ineffective HD-CTX and thus, reduce toxicity and treatment costs in these patients.
